# Assessing the impact of *Samanea tubulosa* trees on methane emissions and its potential as a feed supplement for ruminants in silvopastoral systems

**DOI:** 10.1007/s10457-025-01231-7

**Published:** 2025-06-24

**Authors:** Simón Pérez-Márquez, Vagner Ovani, Vanderson Eliel Meira, Alexandre de Azevedo Olival, Helder Louvandini, Jozivaldo Prudencio Gomes de Morais, Mariana Campana, Tiago Antônio Del Valle, Rogério Martins Mauricio, Adibe Luiz Abdalla

**Affiliations:** 1 Laboratório de Nutrição Animal, Centro de Energia Nuclear Na Agricultura, Universidade de São Paulo, Piracicaba, Sao Paulo, 13400-970 Brazil; 2 Rothamsted Research, Okehampton, Devon, EX202SB UK; 3 Faculdade de Ciencias Biologicas E Agrarias, Universidade Do Estado de Mato Grosso, Alta Floresta, MT 78580-000 Brazil; 4 Departamento de Biotecnologia E Produção Vegetal E Animal Do CCA, Universidade Federal de São Carlos, Araras, São Paulo, 13600-970 Brazil; 5 Departamento de Zootecnia, Universidade Federal de Santa Maria, Santa Maria, RS 97105-900 Brazil; 6 Departamento de Biotecnologia, Universidade Federal de São João del Rei, São João del Rei, MG 36301-160 Brazil

**Keywords:** Ruminants, Greenhouse gases, Sustainability, Native trees, Bordão de velho

## Abstract

Silvopastoral systems (SPS) have been recognized for their multiple benefits, including animal welfare, performance, greenhouse gas mitigation, forage quality, and overall ecosystem services. However, the availability of native tree components for SPS is limited, leading to the prevalent use of exotic species. This study aimed to investigate the potential of the native leguminous tree, *Samanea tubulosa*, in SPS for sustainable livestock production. Chemical-bromatological analysis of *S. tubulosa* fruits revealed a high crude protein content and non-structural carbohydrates (NSC). An in vivo assay with Dorper lambs showed that increasing levels of *S. tubulosa* in diets had a linear effect on both neutral detergent fiber (NDF) and NSC intake, with reduced NDF digestibility. However, no significant effects were observed on dry matter, organic matter, or crude protein digestibility. Moreover, methane (CH_4_) emissions decreased with increasing *S. tubulosa* inclusion, with daily CH_4_ emissions being up to 39% lower in animals fed with *S. tubulosa* compared to those without. Dendrometric characteristics of *S. tubulosa* trees indicated an average height of 8.79 m and a canopy area of 92.04 m^2^ when in pasture, with an average fruit production of 56 kg per tree, allowing for the inclusion of 108 trees per hectare. The study highlights the potential of *S. tubulosa* as a natural feed supplement for ruminants in SPS during the dry season, offering environmental and economic benefits. The integration of this native species into grazing systems can promote sustainable livestock production practices while contributing to the conservation of Brazilian biomes.

**Supplementary Information:**

The online version contains supplementary material available at 10.1007/s10457-025-01231-7.

## Introduction

Silvopastoral systems (SPS), which integrate trees, pastures, and livestock, offer a promising strategy for improving the sustainability of livestock production. Numerous environmental, social, and economic benefits have been reported as a result of adopting SPS. Including, improved animal welfare (Deniz et al. 2023), enhanced animal performance (Lemes et al. 2021), mitigation of greenhouse gas (GHG) emissions (Resende et al. 2020), and increased forage production and quality (Gomes et al. 2020, 2022). Additionally, SPS contribute to improvements in soil chemical attributes, biological diversity, and soil health (Silva-Olaya et al. 2022; Moreno-Galván et al. 2023), as well as positive changes in insect fauna (Paiva et al. 2020). It also provides farmers with shade, firewood, timber, fruits, and live fences (Ortiz Timoteo et al. 2023), along with overall ecosystem services (Torralba et al. 2016; Guimarães et al. 2023), making them a key tool for sustainable agricultural development in regions like Brazil, where land degradation is a pressing concern.

Brazil’s vast and diverse biomes offer a wide array of native tree species with the potential to contribute to SPS. Despite this, the use of exotic tree species remains widespread in Brazilian SPS. This reliance on exotic species is driven by several factors, including well-established cultivation practices, the availability of planting materials, and their effectiveness in certain farming systems (Santos and Grzebieluckas 2014; Huertas et al. 2021). However, the dominance of these species suggests an underutilization of native species, which could provide additional ecological and economic benefits, particularly for small-scale and resource-limited farmers.

Supporting this observation, a search in the Scopus database for the terms “Silvopastoral Systems” and “Brazil” revealed that of 117 studies on SPS in Brazil, 91 focused on exotic species, while only 47 investigated native species. Notably, Eucalyptus and Acacia were the most frequently studied species, while native species remain underrepresented in research and practice (Suppl. 1). This imbalance highlights the need to explore the potential of native trees, not only to reduce the dependence on exotic species but also to harness the unique advantages that native species offer in terms of adaptability and sustainability.

The use of native trees in SPS can provide important ecosystem services while also offering practical benefits to farmers. Many native species are well-adapted to local conditions and can naturally regenerate within pastures, reducing the need for costly inputs and management practices (Esquivel et al. 2008; Murgueitio et al. 2011). Additionally, native species often produce fruits and leaves that can serve as valuable supplements to ruminant diets, especially during the dry season when forage availability is low (Ovani et al. 2023). This can enhance the resilience of the farming system (Balehegn 2017), particularly in regions where conventional forage resources are insufficient to meet livestock needs throughout the year. Olival et al. (2022) identified that family farmers in the southern region of the Amazon recognize various ecosystem functions associated with native tree species present in pastures, and that this perception is fundamental to their decision to keep trees in pastures.

One such native species is *Samanea tubulosa* (Bentham) Barneby & J.W.Grimes, a leguminous tree commonly found in pastures across northern Brazil (Olival et al. 2022). Known locally as “Bordão de Velho,” *S. tubulosa* occurs naturally in several Brazilian states and extends to parts of Argentina, Bolivia, and Paraguay (Carvalho 2007). The species is valued for its ability to restore degraded areas and provide high-quality wood for energy and furniture production (Almeida et al. 2022). More importantly for livestock systems, *S. tubulosa* produces sweet, highly palatable fruits that are readily consumed by cattle, and its foliage is rich in crude protein, making it an attractive fodder resource (Carvalho 2007).

Given its adaptability to local conditions and its potential to supplement ruminant diets, *S. tubulosa* holds promise as a key species for inclusion in SPS. However, to fully understand its value in these systems, it is necessary to evaluate specific traits that influence its contribution to both the environment and animal productivity. Dendrometric characteristics, such as tree height, canopy cover, and growth rate, are important indicators of how well a species can provide shade, timber, and other farm resources. It is also important for determining planting strategies and the arrangement of trees in pastures. Fruit production is equally critical, as it determines the availability of supplemental feed for livestock during times of scarcity. Additionally, the nutritional quality of *S. tubulosa* fruits, particularly in terms of protein and energy content, must be assessed to determine their potential to improve animal performance.

Another key aspect of this study is to explore the impact of *S. tubulosa* on methane (CH_4_) emissions from ruminants. The *S. tubulosa* fruits’s sweetness and palatability could be an indicator of a high content of non-structural carbohydrates which may have the potential to modify rumen fermentation in ways that could mitigate CH_4_ production. By evaluating the CH_4_-reducing potential of *S. tubulosa*, this study contributes to the broader goal of developing low-emission livestock systems.

This study integrates an assessment of tree productivity, fruit nutritional value, and in vivo impacts on methane emissions to explore the role of *Samanea tubulosa* in sustainable silvopastoral systems. By combining dendrometric measurements with feeding trials, we aim to provide practical insights into how this native species could contribute to forage supplementation and climate mitigation, particularly during the dry season when feed shortages are most acute.

Thus, the objectives of this study are threefold: (1) to evaluate the dendrometric characteristics and fruit production of *S. tubulosa*, (2) to assess the nutritional value of its fruits and their effect on sheep intake, and (3) to investigate the potential of *S. tubulosa* to reduce CH_4_ emissions from livestock. Specifically, we hypothesize that (4) the inclusion of fruits of *S. tubulosa* in sheep diets do not negatively affect animal intake and that (5) the inclusion promotes ruminal CH_4_ mitigation. By addressing these key aspects, this study aims to provide a comprehensive understanding of the role that *S. tubulosa* can play in enhancing the sustainability of SPS.

## Materials and methods

### Site description

**Fig. 1:**
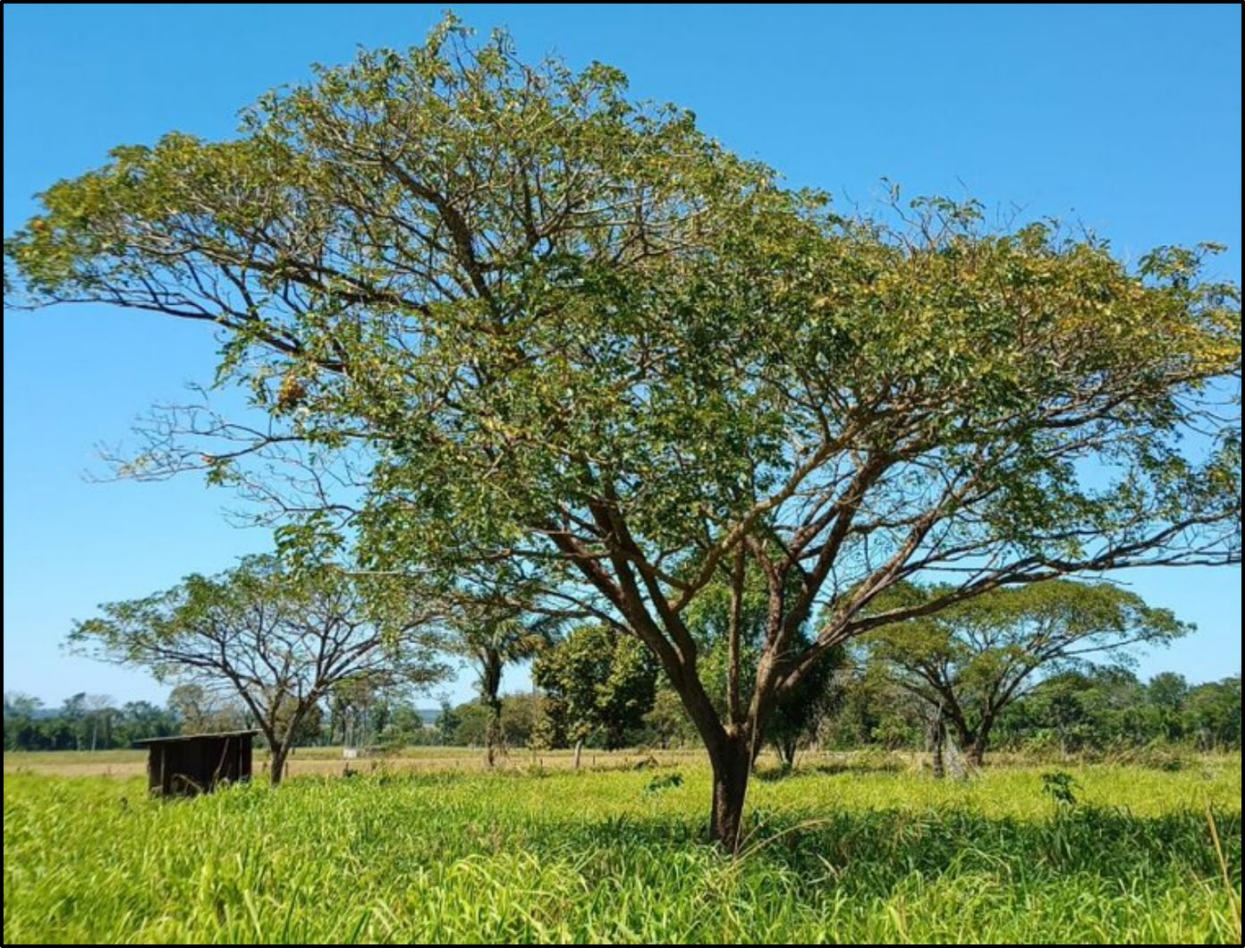
*S. tubulosa* tree in the pasture

For the subsequent procedures described, 60 *S. tubulosa* trees were selected from pasture areas (Fig. 1), agroforestry orchards, and regeneration areas, all located in the northern region of the state of Mato Grosso, Brazil. This region is part of the Amazon biome and is characterized by dense forests and savannas (Moraes R. et al. 2021).

### Tree measurements

The total height and stem height of the trees were measured using a Blume Leiss hypsometer, following the methodology proposed by Feliciano et al. (2016). The estimation of tree age was performed by collecting growth rings with the aid of a Pressler increment borer. A sample was taken from each tree through an incision in the trunk, specifically at breast height (1.30 m above the ground) (Li et al. 2023).The canopy area was determined by measuring 8 radii, forming 8 triangles. The total area was obtained by summing the area of each of the triangles (Costa et al. 2016). The radii were measured using a measuring tape, following the compass orientation, starting from the North (N) position and the other cardinal positions: northeast (NE), east (E), southeast (SE), south (S), southwest (SW), west (W), and northwest (NW) (REX et al., 2018). The circumference at breast height (CBH) was obtained by measuring the circumference of the tree trunk at 1.30 m above the ground (Li et al. 2023).

### Sample and data collection

**Fig. 2:**
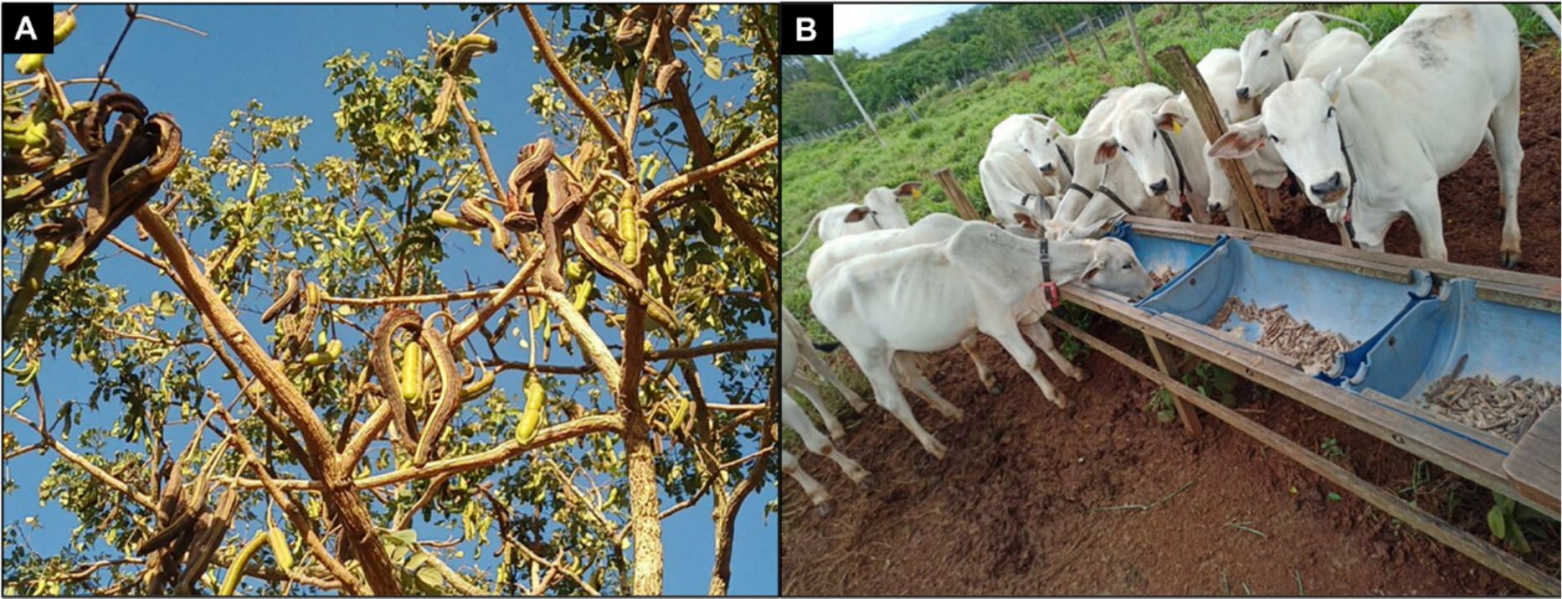
**a** Mature *S. tubulosa* fruits; **b** *S. tubulosa* fruits being used for bovine supplements by small farmers

**Fig. 3:**
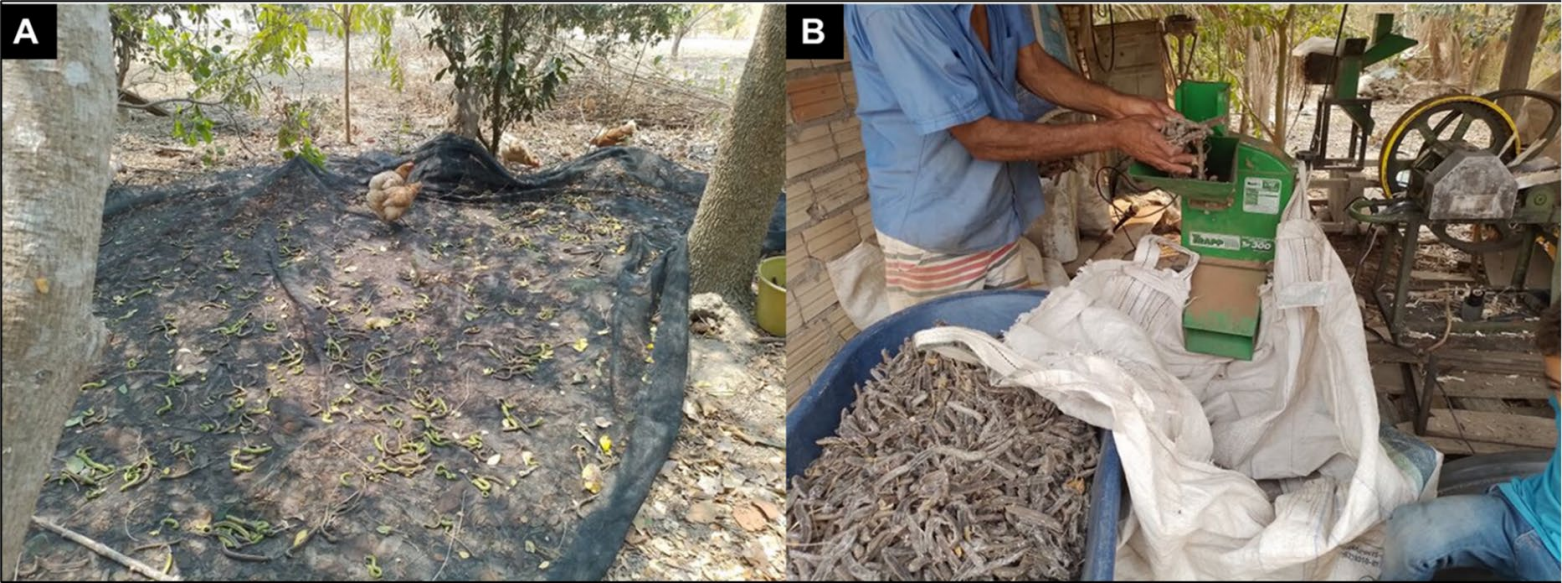
**a** Sun drying and **b** grinding of *S. tubulosa* fruits

We aimed to replicate the procedures that farmers who empirically use *S. tubulosa* fruit to feed their animals already follow (Fig. 2a, b and 3a, b), while standardizing the collection procedures to achieve greater uniformity in the quantity of fruit used in the experiment. Thus, for bromatological analysis and the in vivo assay, *S. tubulosa* fruits were collected at a ripe stage (Fig. 2a) during the dry season (i.e., August to October), when animals typically consume them. The fruits were manually collected by local small farmers who already use them for seed collection and ruminant feeding (Fig. 2b). The fruits were sun-dried (Fig. 3a), stored in plastic drums until further use, and then ground (Fig. 3b) in a forage crusher (Model TRF 300; TRAPP, Jaraguá do Sul, SC, Brazil) to be offered to the animals. A fraction of all collected samples was dried in a forced-air oven (Model MA 037; Marconi, Piracicaba, SP, Brazil) at 55 °C for bromatological analysis and at 40 °C for total and condensed tannin determination. All samples were ground to a 1 mm mesh size using a knife mill and stored at − 20 °C for subsequent analyses.

### Bromatological analysis

The *S. tubulosa* fruit samples were thawed in the open air, and following protocols approved by the Association of Official Analytical Chemists (AOAC 2011), the concentrations of dry matter (DM; Nº: 934.01), crude protein (CP; Nº: 2001.11), ether extract (EE; Nº: 2003.5), and ash (Nº: 942.05) were determined. The contents of neutral detergent fiber (NDF; assayed with heat-stable amylase and expressed exclusive of residual ash), acid detergent fiber (ADF; expressed exclusive of residual ash), and lignin (determined by solubilization of cellulose with sulfuric acid) were determined according to the methodology described by Van Soest et al. (1991) and adapted by (Mertens et al. 2002). The NSC content was estimated using the equation described by Van Soest et al. (1991):

$$NSC = 100 - NDF + CP + EE + Ash$$

where, NSC represents non-structural carbohydrates (g kg^−1^); NDF is neutral detergent fiber (g kg^−1^); CP is crude protein (g kg^−1^); and EE is ether extract (g kg^−1^).

### Animals and experimental diets

The in vivo assay for determining digestibility and GHG emissions was conducted partly at the Center of Agricultural Sciences, of the Federal University of São Carlos, Brazil (i.e., animal adaptation to diets with *S. tubulosa* fruit inclusion), and partly at the Laboratory of Animal Nutrition, Center for Nuclear Energy in Agriculture, University of São Paulo, Piracicaba, Brazil (i.e., determination of digestibility and GHG emissions). All the procedures involving animals were approved by the Ethics Committee of the Center for Nuclear Energy in agriculture (CENA), university of São Paulo (USP).

Eight Dorper lambs (27,39 ± 3.35 kg of BW) were randomly paired and randomly assigned to 4 dietary treatments in a 4 × 4 latin square design with 4, 25-day experimental periods. The first 15 days of each period were used for diet adaptation, followed by a 6-day period of digestibility assay (that is not included in the present work) after which the lambs were put in individual respiration chambers for four days for CH_4_ emissions measurements.

Four diets were formulated with increasing levels of *S. tubulosa*. A base diet composed of corn silage and concentrate (soybean meal and ground corn) and 3 diets with increasing levels of *S. tubulosa* as DM inclusion (6, 12 and 24%) replacing mainly corn silage (Tables 1 and 2). During the evaluating period, each morning before feeding all ingredients from each diet, were individually weighed and mixed for each feeding period to ensure accuracy in dietary inclusion levels.

**Table 1:** Chemical composition of *S. tubulosa* and corn silage

Variables	*S. tubulosa*	Corn Silage	Concentrate
Chemical Composition (g kg^−1^ on DM basis)			
Crude Protein	153.45	97.75	182.68
Neutral Detergent Fiber*	389.38	767.16	270.17
Acid Detergent Fiber**	251.71	425.91	69.88
Lignin **	79.19	73.98	8.33
Ether Extract	5.02	23.98	27.58
Ash	33,28	55.36	59.56
Non-Structural Carbohydrates	418.86	55.75	450.9

^*^Assayed using heat stable amylase and expressed as free of residual ash

^**^expressed as free of residual ash

**Table 2:** Ingredient proportion and chemical composition of four diets with increasing levels of *S. tubulosa* (ST)

Variables	0 ST	6 ST	12 ST	24 ST
Ingredients Proportion (g kg^−1^)				
Corn Silage	947.45	883.70	787.11	653.17
Concentrate	52.55	48.31	53.72	62.06
*S. tubulosa*	0.00	68.00	159.17	284.77
Diet Composition (g kg^−1^ DM)				
Dry matter (g kg⁻ ^1^)	345.45	365.15	443	488.47
Crude Protein	147.91	154.36	156.68	155.19
Neutral Detergent Fiber*	680.25	594.72	543.75	502.53
Acid Detergent Fiber**	314.2	290.32	260.88	251.1
Lignin**	62.88	60.45	72.78	70.77
Ether Extract	26.49	26.84	23.63	22.55
Ash	55.48	57.17	51.49	48.12
Non-Structural Carbohydrates	89.87	166.92	224.45	271.61

^*^Assayed using heat stable amylase and expressed as free of residual ash

^**^expressed as free of residual ash

### Methane measurements

For four days the lambs were allocated into individual respiration chambers equipped with trays for feces collection (Abdalla et al. 2012). Two days for adaptation to the chambers and two days for measurements and sample collection. Air from the chambers was removed via an exhaust pump and sampled into 5-L aluminum balloons using a peristaltic pump in 23-h cycles. Temperature and humidity were measured at regular intervals for each chamber.

After each 23-h cycle of measurement, feed leftovers and complete feces were collected, weighted and dried at 60 °C. The air from the balloons was sampled into 160 mL glass bottles for CH_4_ concentration determination through gas chromatography following the chromatographic conditions described by Lima et al. (2018).

### Feed intake and digestibility

During the 4-day period that the sheep were in the respiration chambers the leftover and feces samples from each animal were collected daily before the first daily meal was provided, weighed on an electronic scale (with a precision of 5 g), sampled 10% (forming a pool of leftovers and a pool of feces for each animal), and stored in a freezer at − 20 °C for subsequent bromatological analysis.

Nutrient’s apparent digestibility was determined according to the equation described by (McDonald et al. 2011).

$$AD of X =\frac{ X\_intake-X\_excreted}{X\_intake}$$

where: AD = Apparent digestibility (g g^−1^); X = evaluated nutrient. Intake was determined daily by the difference between feed offered and feed leftovers.

### Statistical analysis

The data were analyzed using R software version 4.1.1 (R Core Team 2023). All data were first checked for normality using Shapiro–Wilk tests and for homogeneity of variances using Bartlett’s test. Additionally, residual plots were examined to verify the assumptions of linearity, independence, and homoscedasticity. The Latin square design accounted for potential confounding from animal and period effects by including animal as a random effect and period as a fixed effect. The following model was used:

$$Yijk = \mu + Si + Pj + Ak + Eijk$$

where, Yijk represents the response variables affected by the inclusion of *S. tubulosa* fruits "i", animal "j", and period "k"; μ is the overall mean; Pj is the effect of period; Ak is the random effect of animal; and Eijk is the random error associated with each observation. A regression analysis was performed to evaluate the effects of the *S. tubulosa* inclusion on digestibility and CH_4_ emissions.

## Results

### Chemical-bromatological composition of S. tubulosa fruits

The description of chemical-bromatological fruits composition of *S. tubulosa* trees are shown in Table 3. The fruits of *S. tubulosa* are observed to have a greater carbohydrate concentration, with the largest proportion being fibrous carbohydrates, followed by a NSC concentration of approximately 397 g kg⁻^1^ DM. Phenolic compounds are also present, with tannins being the most prominent, primarily in the form of condensed tannins.

**Table 3:** Chemical-bromatological composition of *S. tubulosa* fruits

Variables	n	Mean	SD	Min	Max
aNDFom (g kg^−1^)	9	409.77	22.46	381.41	447.54
NSC (g kg^−1^)	9	396.92	29.64	335.79	432.49
ADFom (g kg^−1^)	9	248.08	16.64	229.69	277.60
CP (g kg^−1^)	9	150.50	10.58	137.87	171.64
Lignin (g kg^−1^)	9	118.41	9.34	106.16	135.26
Ash (g kg^−1^)	9	36.94	2.82	33.11	42.55
EE (g kg^−1^)	9	5.87	1.53	4.10	8.58
Total phenolic compounds^d^	9	70.16	7.21	58.73	78.80
Total tannins^d^	9	58.26	6.73	47.42	66.55
Condensed tannins^e^	9	51.88	7.61	38.71	62.85

DM, dry matter; CP, crude protein; EE, ether extract; aNDFom, neutral detergent fiber assayed with a heat stable amylase and expressed exclusive of residual ash; ADFom, acid detergent fiber expressed exclusive of residual ash; Lignin, determined by solubilization of cellulose with sulfuric acid; NSC, nonstructural carbohydrates; ^d^Values expressed in equivalent gram of tannic acid/kg dry matter; ^e^Values expressed in equivalent gram of leucocyanidin/kg dry matter. SD, standard deviation

### Intake and nutrient digestibility

No significant effects of the *S. tubulosa* inclusion were observed on the intake of dry matter, organic matter nor crude protein (*p* > 0.05). However, a linear effect was observed on both NDF and NSC intake because of the *S. tubulosa* inclusion in the diets (Table 4). For every unit (g kg^−1^) of *S. tubulosa* that increased in the diet, the daily NDF intake reduced in 2.8 g (*p* < 0.001; R^2^ = 0.17) and the daily NSC intake increased in 4 g (*p *< 0.001; R^2^ = 0.29).

**Table 4:** Intake and digestibility coefficients of diets with increasing levels of *S. tubulosa* (ST)

	0 ST	6 ST	12 ST	24 ST	SEM	L	Q	R^2^
Intake (g d^−1^)								
DM	723.8	864.89	840.74	780.5	41.348	0.212	0.098	
OM	682.6	781.76	797.71	742.44	33.95	0.179	0.109	
CP	109.5	130.11	132.3	119.03	8.535	0.256	0.250	
NDF ^a^	478	487	442	394	225	< 0.001	0.176	0.17
NSC ^b^	80.63	138.77	178.29	210.24	21.83	< 0.001	0.761	0.29
Apparent Digestibility Coefficients								
DM	0.73	0.73	0.67	0.70	0.020	0.146	0.207	
OM	0.75	0.75	0.68	0.71	0.187	0.091	0.159	
CP	0.60	0.63	0.56	0.59	0.031	0.802	0.516	
NDF ^c^	0.67	0.62	0.51	0.49	0.023	< 0.001	0.579	0.40

n = 16; SEM = Standard error of the mean; L, Q: *p*-values for the linear and quadratic responses respectively; R^2^ = Determination coefficient. Equations: ^a^Y = 499–2,80x; ^b^Y = 73,2 + 4,08 × ^c^Y = 0,67–0,005x; DM = Dry matter; OM = Organic Matter; CP = Crude protein; NDF = Neutral detergent fiber; NSC = Non-Structural Carbohydrates

There was no effect of the *S. tubulosa* inclusion on the dry matter, organic matter nor crude protein digestibility of the diets. Nonetheless, the NDF digestibility reduced with the increasing inclusion of *S. tubulosa* in the diet (*P *< 0.001; R^2^ = 40). For every increasing unit (g kg^−1^) of *S. tubulosa* in the diet, the NDF digestibility coefficient reduced in 0.5%.

### Methane emissions

The animals fed with the diets with inclusion of *S. tubulosa* emitted less CH_4_ both in absolute terms (g d^−1^) as well as in relative terms (g kg^−1^ DMI and OMI). A linear decrease was observed in the CH_4_ production consequence of the *S. tubulosa* increase in the diet when emissions were expressed as grams per day, per kg of dry matter intake, per kg of organic matter intake and per kg of both digested dry mater and organic matter (Table 5.).

**Table 5:** Methane emissions of sheep fed with increasing levels of *S. tubulosa* (ST)

Methane Emissions (g)	0 ST	6 ST	12 ST	24 ST	SEM	L	Q	R^2^
Per day	7.27	7.18	5.15	4.37	0.754	0.005	0.679	0.12
Per kg of LW	0.60	0.59	0.43	0.38	1.005	0.007	0.744	0.11
Per kg of DMI ^a^	9.87	8.75	6.67	5.54	1.010	0.002	0.996	0.13
Per kg of OMI ^b^	10.47	9.26	7.04	5.82	1.060	0.001	0.995	0.14
Per kg of DDM ^c^	13.35	12.40	9.91	8.78	1.615	0.021	0.980	0.08
Per kg of DOM ^d^	13.81	12.83	10.29	9.11	1.707	0.022	0.981	0.08

n = 16; ST = *Samanea tubulosa*; SEM = Standard error of the mean; L, Q: p-values for the linear and quadratic responses respectively; R^2^ = Determination coefficient; Equations: ^a^Y = 9,96–0,125x; ^b^Y = 10,57–0,134x; ^c^Y = 13,58–0,137x; ^d^Y = 13,97–0,141x; DMI = Dry matter intake; OMI = Organic matter intake; DDM = Digested dry matter; DOM = Digested organic matter

The daily CH_4_ emissions (g d^−1^) of the animals fed with the 24 *S. tubulosa* diet was 39% lower than that of the animals fed without *S. tubulosa*. For every g per kg of *S. tubulosa* in the diet, the CH_4_ emissions (g kg^−1^ DMI) reduced 1.26%.

### Tree parameters of S. tubulosa and their relationship with forage

**Fig. 4:**
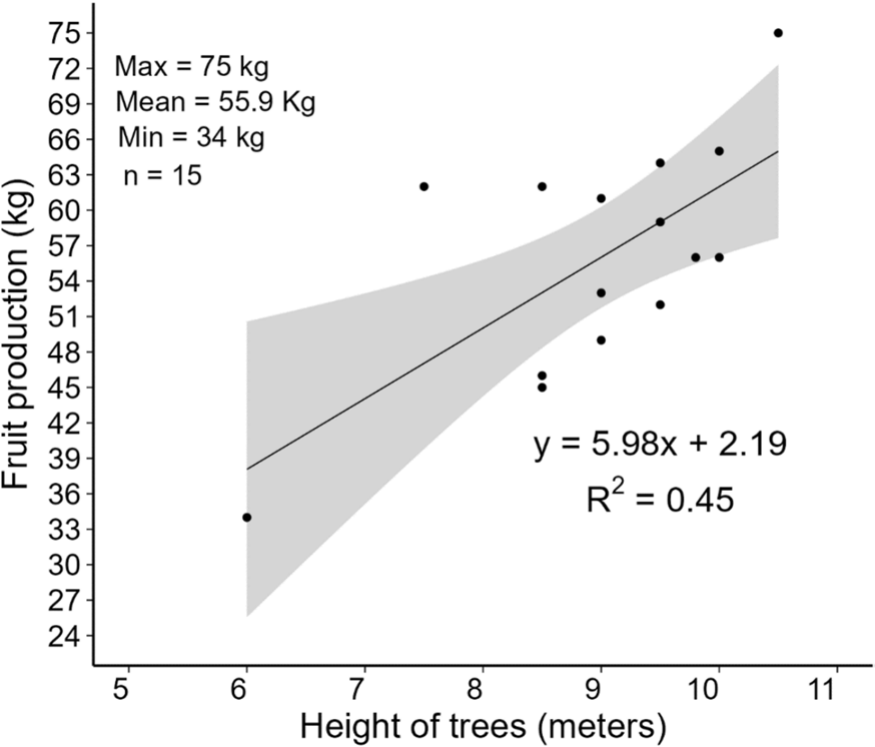
Relationship between fruit production and the height of *S. tubulosa* trees

The description of the dendrometric characteristics of *S. tubulosa* trees is presented in Table 6. The ring test indicates that the trees analyzed in this study were approximately 22 years old, with an average height of 8 m and the presence of bifurcations. A larger canopy area was observed in trees present in pastures compared to those in agroforestry orchards and regeneration areas. A linear relationship was observed between fruit production and the height of *S. tubulosa* trees (Fig. 4), with an average production of 56 kg per tree.

**Table 6:** Dendrometric characteristics of *S. tubulosa* trees in pastures and other areas

Location site	n	Tree age (years)	Height (m)	Shaft height (m)	CBH (m)	Bifurcations number	Canopy area (m^2^)
Pasture	51	22.82 a	8.79 a	2.15 a	0.92 a	1.33 a	92.04 a
Other areas	9	17.78 b	7.54 a	1.58 a	0.61 b	1.22 a	42.85 b
Average	–	22.05	8.60	2.07	0.87	1.32	84.67

^*a,b*^Different letters for different means by the Kruskal–Wallis test; CBH = Circumference at breast height; Other = *S. tubulosa* trees analyzed in agroforestry orchards and regeneration areas

## Discussion

### Tree parameters of S. tubulosa and their relationship with forage

The dendrometric parameters of *S. tubulosa* were evaluated to assess its morphological characteristics and potential effects in a SPS with traditional forage grasses. It can be observed that when found in pastures, the trees have an average height of 8.79 m (Table 6), which classifies them as medium-sized trees according to Andrade et al. (2012). Additionally, following the classification by these authors, *S. tubulosa* has a Flabelliform-shaped canopy (Fig. 1), which is common among tree legumes. These pieces of information are highly relevant when planning the spatial arrangement of the trees, plant distribution, spacing, and plant density per area, as these characteristics can affect the shade intensity and the effects on grass biomass production. In addition to being medium-sized with a Flabelliforme-shaped canopy, *S. tubulosa* has a low canopy density (Fig. 2A), which suggests that it may not significantly hinder light transmission to the biomass of the forage underneath its canopy.

It was also observed that the trees present in the pastures have a higher average age, CBH, and canopy area compared to the trees found in other areas. These differences can be attributed to factors such as resource availability, competition with other species, and management practices adopted in the pastures. In fact, some tree species, when present in isolation in pasture areas, tend to prioritize canopy growth due to the absence of competition and abundance of light. In the case of the studied species, given its low canopy density and favorable architecture for intercropping with forage plants, the larger canopy area may be related to higher fruit production and greater positive effects on the soil and forage plant (Olival et al., 2021; Pang et al., 2019).

### Chemical-bromatological composition of S. tubulosa fruits

Our first hypothesis in this study revolves around the potential negative effects on intake and rumen function consequence of the presence of secondary metabolites in the *S. tubulosa*. Special attention is given to the effects of tannin concentrations, particularly condensed tannins, which, at concentrations exceeding 50 g kg⁻^1^ DM, may affect nutrient intake and digestion, while at lower concentrations can improve protein utilization and reduce CH₄ emissions during enteric fermentation (Ovani et al. 2023). We observed that our fruits of *S. tubulosa* contained concentrations of approximately 51.88 g kg⁻^1^ DM of condensed tannins, which is near the threshold. However, considering that the fruits were incorporated as part of a mixed diet, the effective tannin concentration consumed by the animals would likely have been lower. This leads us to believe that this concentration would not significantly affect intake and may contribute to CH_4_ mitigation. Further research is needed to evaluate the long-term effects of *S. tubulosa* tannins on rumen health and performance under practical conditions. 

Additionally, based on the descriptions provided by Carvalho (2007), which describes *S. tubulosa* fruit as fleshy, with sweet, fragrant pulp and a sweet flavor, we hypothesize that *S. tubulosa* fruits have great potential as an energetic and protein-rich supplement. This was confirmed by our results, which indicate a NSC and a CP content of around 396 and 150 g kg⁻^1^, respectively, surpassing the CP content typically found in commonly used roughage feedstuffs in Brazil (e.g., corn silage, Marandu grass, Mombaça grass) (Table 7). It is important to note that these fruits are produced during the dry season when forage plants reach their lowest protein concentration, often below 7% CP (Tambara et al. 2021).

**Table 7:** Crude protein (CP) and Neutral Detergent Fiber (NDF) composition of forages and supplements commonly used in Brazil

Feed	CP	NDF	References
	g kg^−1^		
Soybean meal	473	37	(Wang et al. 2011)
	511	121	(Moraes et al. 2016)
	482	85	(Ravindran et al. 2014)
Corn silage	81	483	(Wernersbach Filho et al. 2006)
	76	503	(Oliveira et al. 2017)
Corn grain	93	167	(Lima et al. 2022)
	81	147	(Moraes et al. 2016)
*Urochloa brizantha cv. Marandu*	80	700	(Rueda et al. 2003)
	139	555	(Pequeno et al. 2015)
	128	731	(Euclides et al. 2008)
	58	697	(Sousa et al. 2010)
*Megathyrsus maximus cv. Mombaça*	126	745	(Euclides et al. 2008)

These results allow us to hypothesize that the inclusion of *S. tubulosa* fruits in the diet of animals grazing predominantly on roughage could potentially benefit rumen function. The fruits provide extra protein and rapidly digestible energy (i.e., higher NSC content), which may stimulate rumen microbial growth and enhance the propionate production pathway (Ovani et al. 2023). This microbial growth not only improves fiber degradation but also increases microbial protein passage. Once digested in the abomasum and intestines, this protein becomes the primary source of metabolizable protein for ruminants (Furlan et al. 2006; Santos 2006).

### *Intake, nutrient digestibility and CH*_*4*_* emissions*

The inclusion of *S. tubulosa* fruit meal in sheep diets demonstrated promising effects on nutrient intake and digestibility, alongside significant reductions in CH_4_ emissions. Diets were formulated to be iso-proteic, as indicated by consistent CP intake, but differences in fiber and carbohydrate composition were notable when compared to corn silage. The *S. tubulosa* fruit meal had a lower NDF content and a higher proportion of NSC, with a more significant fraction of ADF relative to its fiber content (64% ADF in *S. tubulosa* vs. 55% in corn silage). As the proportion of *S. tubulosa* in the diet increased, there was a linear reduction in NDF intake and digestibility, likely due to the higher NSC content.

The reduction in fiber digestibility with *S. tubulosa* supplementation can be attributed to the well-known detrimental effects of NSC on fiber degradation in the rumen. NSC supplementation shifts the ruminal microbial population from fibrolytic to amylolytic organisms, decreasing fiber breakdown (Souza et al. 2010; Costa et al. 2011). Despite this, dry matter, organic matter, and CP intake and digestibility remained unaffected, suggesting that the inclusion of *S. tubulosa* fruit meal did not compromise overall feed intake.

Methane emissions were significantly reduced with the replacement of corn silage with the *S. tubulosa* fruit meal, showing a 39% reduction in diets containing 24% *S. tubulosa*. This reduction can be explained by multiple mechanisms. First, the shift in ruminal SCFA production towards more propionate, driven by the higher NSC content in the diet, likely played a major role. Propionate acts as a hydrogen sink, diverting hydrogen away from methanogens, and thereby lowering CH_4_ production (Getachew et al. 1998; Amanzougarene and Fondevila 2020). At the same time, this shift likely decreased acetate and butyrate pathways, which are hydrogen-producing routes, further lowering the substrates available for CH_4_ synthesis.

Additionally, forage-based diets with higher structural fiber content typically produce more CH_4_ per unit of digested feed than those richer in grains due to the lower degradability of cell wall components like cellulose and hemicellulose (Sniffen et al. 1992; Janssen 2010; Hua et al. 2022). The reduction in CH_4_ emissions observed here aligns with the higher NSC and lower fiber content in diets incorporating *S. tubulosa*, which is consistent with findings that lower fiber or higher NSC diets lead to less CH_4_ production (Blümmel and Bullerdieck 1997; Janssen 2010).

Another factor contributing to CH_4_ reduction could have been the presence of tannins in *S. tubulosa* fruits. Low concentrations of condensed tannins are known to inhibit CH_4_ production, either through direct action on methanogenic microorganisms or by indirectly affecting hydrogen pathways (Tavendale et al. 2005; Moreira et al. 2013; Lima et al. 2018). Further experiments specifically designed to evaluate the effects of *S. tubulosa*’s tannins on rumen methanogenesis would be valuable.

### Potential and challenges of including S. tubulosa in silvopastoral systems

The nutritional value and the results of the in vivo trial indicated a strong potential for *S. tubulosa* fruits to serve as a natural supplement in tropical SPS, with the ability to mitigate CH₄ emissions without significantly altering feed intake and diet digestibility in animals. The promotion of pasture-based production systems with GHG mitigation potential is aligned with the recent findings of Wang et al. (2024), who discovered that pasture-finished beef had lower GHG emissions per economic activity compared to feedlot-finished beef, emphasizing the need to incorporate land use and management practices, as well as soil carbon sequestration, as fundamental aspects of GHG emissions assessments and mitigation strategies for beef production.

*Samanea tubulosa* is commonly found in pasture areas of the Amazon biome due to natural regeneration processes. However, for its inclusion in traditional SPS arrangements with trees distributed in rows, it is important to evaluate tree density per hectare and its effects on forage production of the grass species.

The morphological characteristics of the canopy of this tree allow us to classify it as suitable for use in SPS. This was also observed by Salman et al. (2012), who, in studying 52 native tree species from Amazonia, indicated the *S. tubulosa* as one of the greatest potential trees for the SPS in the region because its morphological aspects and ecological and economical services.

In our in vivo experiment, lambs on the 24% *S. tubulosa* diet consumed an average of 11.8 (± 2.49) g of fresh *S. tubulosa* fruit meal per kg of live weight (LW) daily. Extrapolating these results to grazing conditions, considering the average fruit production per tree of 55.9 kg (Fig. 4) and a consumption rate of 11.8 g of *S. tubulosa* fruit per kg of LW, an animal weighing 450 kg would require 5.3 kg of fruit meal per day. It is important to note that fruit yield may vary between years due to climatic factors, soil conditions, and tree maturity, which could affect the consistency of supply for supplementation purposes.

In a meta-analysis conducted by de Oliveira et al. (2022), which evaluated traditional SPS with *Eucalyptus* and *Urochloa*, it was observed that most studies used between 200 and 399 trees per hectare. However, they found that forage mass decreased in SPS with more than 99 trees per hectare. Considering that the average canopy area of *S. tubulosa* is 92 m^2^ (Table 6), 99 trees would cover 91% of one hectare, which is impractical due to the shading effects on the pasture. Thus, based on the literature and the results of the study, we can plan to use *S. tubulosa* in two different ways: as a method of afforesting pastures, covering 20 to 30% of the pasture area, or as an orchard exclusively aimed at fruit collection for animal feed. In the first case, according to the average canopy area of *S. tubulosa*, 21.7 to 32.6 trees would be required to cover 20 to 30% of one hectare of pasture. Estimating the presence of around 25 trees per hectare would generate a fruit production of 1,398 kg per hectare per year, which, considering the need for 5.3 kg of fruit meal per day and a supplementation period of 120 days, could feed 2.2 animals per hectare. In the case of fruit orchards, one hectare can have 108 *S. tubulosa* trees, with the potential to produce 6,037.2 kg of fruit, which could feed 9.5 animals for 120 days, considering the inclusion of 24% *S. tubulosa* fruit in the diet. While this extrapolation provides a conceptual approximation of potential supplementation levels, further on-farm research is required to validate these assumptions under practical grazing conditions.

It is important to highlight that the use of *S. tubulosa* can contribute to both soil fertility, as it is a nitrogen-fixing legume, and to the nutritional quality of forages. By incorporating *S. tubulosa* into grazing systems, farmers can reduce their reliance on external inputs, such as commercial feeds and fertilizers, while simultaneously enhancing the sustainability of their production systems. The use of the fruit as part of a balanced diet may also represent an important advance in terms of reducing costs, increasing productivity and reducing the environmental impacts of the activity, especially during the period of forage scarcity.

The challenges of using *S. tubulosa* in SPS are related to the lack of basic knowledge when compared to traditional tree resources. Therefore, it highlights the importance of developing studies aimed at evaluating and reducing the growth period of these trees, as well as assessing other factors such as propagation methods and cultural practices.

Beyond the nutritional and environmental findings, this study offers practical considerations for integrating *S. tubulosa* into pasture systems by linking field data on fruit yield and canopy characteristics with animal supplementation needs. This approach may help inform planning decisions for farmers interested in using naturally regenerating species for seasonal supplementation.

It is important to acknowledge that, although the 4 × 4 Latin square design employed in this study is widely accepted for nutritional evaluations with small ruminants, the limited sample size could restrict broader extrapolations. Future studies involving larger populations would help to confirm and expand upon the findings reported here.

## Conclusion

*Samanea tubulosa* trees produce an average of 55.9 kg of fruit. The fruits have a high concentration of CP and NSC, along with low tannin concentrations, making them a valuable natural supplement for grazing ruminants during the dry season. The inclusion of *S. tubulosa* in sheep diets has been shown to significantly reduce CH_4_ emissions, with the inclusion of 24% *S. tubulosa* fruit providing the best performance in terms of CH_4_ mitigation. However, attention should be given to formulating an adequate NDF level to avoid impairing fiber digestibility. Overall, this study provides valuable insights into the potential of *S. tubulosa* as a feed supplement for ruminants in SPS and its impact on GHG emissions. The findings underscore the importance of incorporating native species into grazing systems, promoting sustainable livestock production practices, and contributing to the conservation of Brazilian biomes. By optimizing resource utilization, improving animal welfare, and mitigating environmental impacts, *S. tubulosa* stands as a promising solution for the enhancement of livestock production in Brazil's diverse agroecosystems.

## Supplementary Information

Below is the link to the electronic supplementary material.

Supplementary file1 (DOCX 19 KB)

Supplementary file2 (XLSX 24 KB)

## Data Availability

All data supporting the findings of this study are available within the paper and its Supplementary Information.
