# Supplementary material for: Assessing the impact of Samanea tubulosa trees on methane emissions and its potential as a feed supplement for ruminants in silvopastoral systems
Source: Agrofor Syst. 2025 Jun 24;99(6):135. doi: 10.1007/s10457-025-01231-7 (PMC12187857; doi:10.1007/s10457-025-01231-7)
Supplement: Supplementary file 1 — Supplementary file1 (DOCX 19 KB) [file 10457_2025_1231_MOESM1_ESM.docx]

**Supplementary Material – Confidence Intervals for Significant Responses**

This document presents the means, standard errors (SE), and 95% confidence intervals (CI) for variables that showed statistically significant effects of *Samanea tubulosa* (ST) inclusion.
Included are tables for digestibility coefficients, nutrient intakes, and methane emissions with significant linear responses (p < 0.05).

**Table S.1 - NDF Intake**

|  | **0 ST** | **12 ST** | **24 ST** | **36 ST** |
| --- | --- | --- | --- | --- |
| Mean (g/day) | 477.63 | 487.42 | 441.83 | 394.32 |
| SE | 22.54 | 22.54 | 23.43 | 22.53 |
| Lower 95% CI | 424.33 | 434.12 | 386.42 | 341.05 |
| Upper 95% CI | 530.93 | 540.72 | 497.24 | 447.59 |

**Table S.2 - NSC Intake**

|  | **0 ST** | **12 ST** | **24 ST** | **36 ST** |
| --- | --- | --- | --- | --- |
| Mean (g/day) | 80.63 | 138.77 | 178.29 | 210.24 |
| SE | 21.21 | 21.83 | 21.20 | 21.20 |
| Lower 95% CI | 30.47 | 87.14 | 128.16 | 160.11 |
| Upper 95% CI | 130.79 | 190.40 | 228.42 | 260.37 |

**Table S.3 - NDF Digestibility**

|  | **0 ST** | **12 ST** | **24 ST** | **36 ST** |
| --- | --- | --- | --- | --- |
| Mean | 0.671 | 0.623 | 0.506 | 0.490 |
| SE | 0.022 | 0.022 | 0.023 | 0.023 |
| Lower 95% CI | 0.618 | 0.569 | 0.451 | 0.436 |
| Upper 95% CI | 0.724 | 0.676 | 0.562 | 0.544 |

**Table S.4 - Methane Emission – g/day**

|  | **0 ST** | **12 ST** | **24 ST** | **36 ST** |
| --- | --- | --- | --- | --- |
| Mean | 7.27 | 7.18 | 5.15 | 4.37 |
| SE | 0.75 | 0.75 | 0.75 | 0.75 |
| Lower 95% CI | 5.49 | 5.39 | 3.37 | 2.59 |
| Upper 95% CI | 9.05 | 8.96 | 6.93 | 6.15 |

**Table S.5 - Methane Emission – g/kg Live Weight**

|  | **0 ST** | **12 ST** | **24 ST** | **36 ST** |
| --- | --- | --- | --- | --- |
| Mean | 0.596 | 0.590 | 0.429 | 0.377 |
| SE | 0.059 | 0.059 | 0.059 | 0.059 |
| Lower 95% CI | 0.458 | 0.451 | 0.290 | 0.238 |
| Upper 95% CI | 0.735 | 0.728 | 0.567 | 0.516 |

**Table S.6 - Methane Emission – g/kg Dry Matter Intake**

|  | **0 ST** | **12 ST** | **24 ST** | **36 ST** |
| --- | --- | --- | --- | --- |
| Mean | 9.87 | 8.75 | 6.67 | 5.54 |
| SE | 1.010 | 1.010 | 1.010 | 1.010 |
| Lower 95% CI | 6.98 | 6.47 | 4.39 | 3.26 |
| Upper 95% CI | 11.65 | 11.03 | 8.95 | 7.82 |

**Table S.7 - Methane Emission – g/kg Organic Matter Intake**

|  | **0 ST** | **12 ST** | **24 ST** | **36 ST** |
| --- | --- | --- | --- | --- |
| Mean | 10.47 | 9.26 | 7.04 | 5.82 |
| SE | 1.06 | 1.06 | 1.06 | 1.06 |
| Lower 95% CI | 7.95 | 6.75 | 4.53 | 3.31 |
| Upper 95% CI | 12.98 | 11.77 | 9.56 | 8.34 |

**Table S.8 - Methane Emission – g/kg Digested Dry Matter**

|  | **0 ST** | **12 ST** | **24 ST** | **36 ST** |
| --- | --- | --- | --- | --- |
| Mean | 13.35 | 12.40 | 9.91 | 8.78 |
| SE | 1.62 | 1.62 | 1.65 | 1.62 |
| Lower 95% CI | 9.53 | 8.58 | 6.01 | 4.96 |
| Upper 95% CI | 17.17 | 16.22 | 13.81 | 12.60 |

**Table S.9 - Methane Emission – g/kg Digested Organic Matter**

|  | **0 ST** | **12 ST** | **24 ST** | **36 ST** |
| --- | --- | --- | --- | --- |
| Mean | 13.81 | 12.83 | 10.29 | 9.11 |
| SE | 1.67 | 1.67 | 1.71 | 1.67 |
| Lower 95% CI | 9.85 | 8.87 | 6.26 | 5.16 |
| Upper 95% CI | 17.76 | 16.78 | 14.33 | 13.07 |
